# Experimental Models of Spinal Cord Injury in Laboratory Rats

**Published:** 2018

**Authors:** A. N. Minakov, A. S. Chernov, D. S. Asutin, N. A. Konovalov, G. B. Telegin

**Affiliations:** Branch of Shemyakin and Ovchinnikov institute of bioorganic chemistry Russian academy of sciences, Prospekt Nauki, 6, Moscow region, Pushchino, 142290, Russia; Federal State Autonomous Institution «N .N. Burdenko National Scientific and Practical Center for Neurosurgery» of the Ministry of Healthcare of the Russian Federation, 4th Tverskaya-Yamskaya Str., 16, Moscow, 125047, Russia

**Keywords:** spinal cord injury, laboratory rat, biomodeling, scaffolds

## Abstract

Pathologies associated with spinal cord injury are some of the leading diseases in the world. The search for new therapeutic agents and 3D biodegradable materials for the recovery of spinal cord functions is a topical issue. In this review, we have summarized the literature data on the most common experimental models of spinal cord injury in laboratory rats and analyzed the experience of using 3D biodegradable materials (scaffolds) in experimental studies of spinal trauma. The advantages and disadvantages of the described models are systematically analyzed in this review.

## INTRODUCTION

One of the most topical and socially significant issues of modern regenerative medicine is the recovery of spinal cord functions in structural defects of various genesis, most of which are caused by injury [1]. Spinal cord injury (SCI) is recognized as one of the main causes of disability [2]. According to the WHO, up to 500,000 people suffer spinal cord injuries annually [3]. The main causes of SCI are road traffic accidents (38%), falls (22.2%), and sports injuries and accidents (22.5%) [4]. The clinical picture of SCI is characterized by a motor activity deficit, impaired sensory and autonomic functions, and neuropathic pain. The pathogenesis of spinal trauma is usually burdened with a poor prognosis associated with the development of paralysis. In addition, some diseases may cause or increase the risk of spinal cord injury [5]. Along with the direct SCIconsequences associated with a loss of motor, sensory, and autonomic functions, there is a risk of secondary processes that may aggravate injury and lead to muscle atrophy, chronic pain, urinary tract infection, and pressure ulcers [6, 7].

Our modern understanding of nerve growth stimulation and immunological, inflammatory, and cicatricial reactions arising in response to SCI has led to the development of several pharmacological treatments. These treatments, in combination with various cellular and additive techniques, bring hope that most spinal cord injuries will be curable in the near future [8-11].

Testing of new materials and techniques that promote regeneration of the spinal cord in animal models is a necessary and important stage in the preclinical development of a strategy for treating spinal cord injuries. One of the key objects used for biomodeling of spinal trauma is the rat. Spinal cord injuries in rats have become the main model used to evaluate the strategy of experimental treatment of SCI [4, 12]. In this review, we describe recent advances in the use of 3D biodegradable materials (scaffolds) designed to provide regenerative growth of axons over the entire injury area of the spinal cord, thereby creating the environment for its endogenous recovery.

## EXPERIMENTAL MODELS OF SPINAL CORD INJURY IN LABORATORY RATS

When choosing the optimal animal model for solving specific research problems, it is necessary to take into account many factors: the type, age, size, and gender of animals and the possibility of using visualization techniques and a functional assessment of their condition. Since the second half of the last century, techniques for the prevention of consequences arising from spinal cord injury have been the subject of systematic studies in various animals, including rats, mice, cats, dogs, and minipigs [13-15]. Experimental models differ in the types of spinal cord injury: contusion, compression, distraction, dislocation, chemical, ischemic, and reperfusion injury, as well as various types of laceration. Of the numerous SCI models developed on rats, the most extensively used models are those relevant to the clinical practice of closed injuries: compression-simulating impaction and contusion-simulating bruise [16-18]. The mean experiment duration in most studies is about 2 months. The main criterion for assessing the adequacy of a model is the detection of morphological changes (axonal regeneration, myelination, vascularization, glial scar density, inflammatory reaction) using histological techniques (usually, transverse and sagittal sections in the injury area and in adjacent (proximal and distal) areas are studied). Auxiliary criteria include MRI diagnostics and electromyography-based functional evaluation. Clinical evaluation is based on the Basso, Beattie, and Bresnahan rating scales (BBB test), with a rat moving inside a plexiglass cage equipped with digital cameras and somatosensory potential registration [19-21], dynamic weight bearing (DVN) test [22], and behavioral tests.

The disadvantages of most experimental rat SCI models are poor control of the impact extent, as well as deep destructive changes in the gray and white matter of the spinal cord, including pathological shifts, death of neurons and glial cells, degeneration of nerve fibers, demyelination, and activation of microglia and macrophages [23]. All these impairments lead to the development of a stable functional deficit. Models of contusion, compression, traction, photochemical, inflammatory, ischemic, and reperfusion injuries have been primarily used for the investigation of the SCI pathophysiology, because they reproduce the potential mechanisms of trauma and spinal cord injury [15]. The presented modeling methods might adequately reflect clinical and morphological shifts in SCI in humans, but most of the models are difficult to reproduce, and they cannot be used to study spinal cord regeneration in structural injuries.

Functional deficit of the spinal cord in rats has been proven to be mainly associated with failure of the conductive white matter tracts [24]. Therefore, the pathophysiological processes of spinal cord injury should be considered analogously to the processes associated with injury to the peripheral nervous system. The dependence of the peripheral nerve ability to restore innervation on the injury extent was established and quantified (as three- and five-point scales) as early as the middle of the last century [25-28].

In the case of mild injury (neuropraxia) to peripheral nerves, axonal regeneration has been experimentally proven and confirmed in clinical practice. There are numerous examples of restoration of effector site innervation in mammals both surgically and spontaneously. Cell-signaling factors were found to arise among neurons, Schwann cells, macrophages, and the environment, which contributes to remyelination, growth, and, which is noteworthy, self-guidance of the regenerating axon [29-32]. Restoration of conduction occurs in several stages, including myelination, axonal growth, formation of synaptic contacts, and, finally, recovery of effector functions [33]. Axonal regeneration has been proven to occur in the rostrocaudal direction, along the former fiber course, with a mean rate of about 1 to 2 mm per day [34-38].

In moderate injuries, the injury site is characterized by axonal demyelination and anterograde (extending from the injury site to the peripheral segment) Wallerian degeneration of the distal nerve coming to the effector, while the proximal nerve and neuronal body remain unaffected, causing, e.g., phantom pain after limb amputation [39].

In severe cases, neuroma and glial scars may develop. Ipsilateral cysts (syringomyelia, cystic degeneration), mainly in the lateral funiculi of the spinal cord white matter, develop in 30% of the total number of clinical cases [40]. At the stage of cicatricial degeneration, glia has been found to perform the barrier function, preventing the spread of histolysis products and inflammatory mediators (mainly macrophages), and also to support the architectonics of central nervous system organs. However, the tissue structure of these defects tightens in the course its formation and prevents regenerative growth of axons, resulting in the fact that the central nervous system axons of adult mammals cannot regenerate spontaneously after an injury associated with demyelination [40-42].

One of the surgical treatment options for the most common form of chronic SCI (at the stage of formed structural defects) requiring surgery is to create favorable conditions for axonal growth by providing “free” space in the structural defect area via the removal of mechanical barriers (scars) by their excision to healthy tissue. This idea has served as the basis for a number of studies on the surgical creation of a structural defect of the spinal cord in rats by complete transection of the cord with a scalpel [43-52] and partial resection with microsurgical scissors [41, 53-57].

Partial transection of the spinal cord (hemisection) enables a comparison of damaged and healthy fibers in the same animal. For example, hemisection may be used to study the locomotor function and its recovery at different levels of the spinal cord, as well as to compare neurological deficits in contra- and ipsilateral lesions. In addition, partial transection of the spinal cord results in a less severe injury compared to complete transection, which largely facilitates postoperative care of animals [58]. Many studies have shown that recovery of the spinal cord function in rats occurs within the first 3 weeks after injury [13, 59], which cannot be attributed solely to the compensatory abilities and regeneration of damaged axons. This also indicates that a unilateral spinal cord injury leads to reversible dysfunction of the spinal cord, because posttraumatic changes in the tissue do not involve the spinal cord areas contralateral to the injury site [60]. It should also be remembered that assessment of the extent of the injury is not always possible. In these cases, researchers have to use somatosensoryevoked potentials to improve the accuracy of their experiments [61].

The complete spinal cord transection model is a dissociation between the caudal and rostral segments of the spinal cord and is easily reproducible. Spinal cord transection is followed by a cascade of complex pathophysiological processes that inhibit potential regeneration of axons and form a glial scar. This model is described in various animals, including rats, mice, cats, dogs, and primates [62]. Thus, the complete spinal cord transection model is most convenient in terms of tissue engineering opportunities [63]. A complex approach to the treatment of SCI using scaffolds that are also able to deliver both target molecules and cells to an injured site of the spinal cord can use only models of partial structural injury of the spinal cord: they are helpful both for the assessment of axonal regeneration and for the subsequent functional recovery.

In most studies, an experimental spinal cord injury is modeled at the thoracic spine level [37, 47, 50-54, 57, 64, 65]. In humans, SCI usually occurs at the cervical level; in particular sports injuries or road accident injuries [48, 49, 55, 56]. In this regard, recent studies have mainly focused on cervical-level-injury models. In these models, compared to thoracic-spinal-cord-injury models, a pronounced neurological deficit develops, complicating the care and observation of animals in the postoperative period and dramatically increasing lethality [66]. Lumbar-level SCI models have been described, but less frequently [67]. However, the neurological deficit caused by a lumbar-spinal-cord injury largely results from damage to the gray matter (most developed in the lumbar enlargement region) rather than from damage to the white matter. Observations demonstrate that gray matter injury may lead to significant functional deficit, including paraplegia, without interruption of the main descent pathways.

## THE USE OF SCAFFOLDS FOR STIMULATION OF REGENERATION AND FUNCTIONAL RECOVERY OF THE SPINAL CORD

The active development of additive technologies of stereolithography and tissue engineering has provided a powerful impetus for the creation of new biocompatible biodegradable three-dimensional scaffold materials capable of stimulating the regeneration of axons and their functional recovery. Most studies in the SCI field are aimed at reducing secondary injuries and promoting tissue regeneration [7]. The most common approach to the treatment of SCI is the combined one that involves scaffolds, cell transplantation, and the delivery of bioactive substances [33, 68].

The main requirement for scaffolds is biocompatibility, which should create an environment that promotes growth and vascularization of tissue and enables axons to regenerate through a graft. A number of research teams have studied biodegradable 3D scaffold materials [7, 49, 65, 69-78]. Honeycomb [47], nanofiber [49], and sponge [50] scaffolds were studied. In this case, many questions related to material biocompatibility arose. Recent studies have quantitatively proven that implantation of scaffolds into the area of a structural defect of the spinal cord contributes to axonal regeneration. For example, in one study, the motor function was recovered one month after microfilament scaffold implantation, and remyelinated nerve fibers were reliably detected in the scaffold structure two months after the completion of the experiment. The fibers amounted to 10–25% of the total amount of conductive pathways [33].

Another direction in the development of scaffolds is the creation of carcasses (hydrogels) with physical properties close to those of tissues [54, 57]. The similarity of the physical properties of an implant and a substrate revealed a 3- to 4-fold increase in the intensity of regenerative axonal growth in hydrogels compared to rigid mechanical scaffolds [37]. Capillary and porous hydrogels were studied in vivo. A characteristic feature of hydrogels noted by the authors was the loss of channel linearity in implants in a chronic experiment [22]. One of the advanced technologies for the production of hydrogel implants is two-photon polymerization. According to the authors, scaffolds produced using this innovative technique minimize injury to the surrounding tissues and provide architectural support to the surrounding tissues during the post-traumatic period, which prevents the destruction of neural networks in the defect area [79, 80].

Along with providing mechanical support and identifying the direction of axonal growth, there are studies that focus on the stimulation of regenerative processes by the bioactive compounds present in scaffold channels. Synergism of the microenvironment with neurotrophic factors has been proven to promote more efficient regenerative processes during the rehabilitation period in a structural injury of the spinal cord [81]. These growth factors include stem cells [7, 42, 44, 82-85], nerve cell growth factors [86-89], and even locally delivered magnetic nanoparticles [90]. Polylactide-co-glycolide multichannel scaffolds containing Schwann cells derived from newborn rats were proposed for directed axonal growth [76]. Placement of these structures in the spinal cord wound of adult rats led to the regeneration of injured axons a month after implantation. Later, replacement of Schwann cells in the scaffold channels with mesenchymal stem cells of the bone marrow led to a similar effect of injured axon regeneration in rats with SCI [83].

The issue of an adequate choice of the channel diameter is of particular importance in the development of multichannel biodegradable scaffolds [48, 56]. In rats, the diameter of axons is known to range from 1 to 8 µm, with a cross section of 2–4 µm being predominant [91, 92]. When creating the structure of internal scaffold channels, it is necessary to take into account the fact that, during regeneration, a new myelin sheath is first formed, through which the axon grows subsequently [93, 94]. For example, an increase in the channel diameters of alginate scaffolds by 50% (from 41 to 64 µm) stimulated the regenerative activity of axons by more than two fold [37].

**Table:** Generalized information on experimental models of SCI in rats

No.	Approach level	Injury	Complexity	degree*	Invasiveness	degree* Application References
1	C2	Left hemisection	+++	+++	Assessment of functional recovery	[20]
2	C4	Resection	+++	+++	Investigation of regenerative processes in the conductive pathways upon scaffold implantation and under the influence of a neurotrophic growth factor	[48, 58]
3	C5	Contusion	++	+++	Study of electro- and pathophysiology of injury	[13, 66]
4	C5	Transverse resection of a spinal cord segment	+++	+++	Investigation of axonal regeneration within the scaffold structure	[37, 65]
5	T3, T3–6	Transverse resection of the spinal cord	+++	+++	Study of motor axon regeneration in fibrin gel under action of neuronal stem cells and growth factor (NGF) within the scaffold structure	[9, 46, 47]
6	T5–7	Compression	+++	++	Assessment of clinical consequences, depending on the time of experimental compression of the spinal cord	[16, 18]
7	T6–7	Transverse resection of the spinal cord	+++	+++	Implantation of scaffolds; investigation of regeneration of injured axons	[83]
8	T6–10	Chemical injury	++	+++	Investigation of nerve fiber remyelination	[32]
9	T7–9, T7–10	Transverse resection of a spinal cord segment	++	+++	Implantation of scaffold; study of the axon ability to grow through the scaffold	[53, 63]
10	T7–12	Complete spinal cord transection	+++	+++	Study of spontaneous recovery of hindlimb mobility after injury	[60]
11	T8	Transverse resection of a spinal cord segment	++	+++	Implantation of scaffolds of different structure	[67, 69, 70, 78]
12	T8–9, T9	Transverse resection of a spinal cord segment	++	+++	Investigation of axonal remyelination within fibrillar collagen scaffolds and the possibility of spontaneous functional recovery	[33, 50, 52, 59]
13	T9	Contusion	++	+++	Assessment of contusion severity by locomotor tests and investigation of the influence of mesenchymal stem cells on regenerative processes	[21, 64]
14	T9	Contusion followed by resection of a glial scar	+++	+++	Replacement of a glial scar with collagen scaffolds with mesenchymal stem cells	[84]
15	T9–10	Hemilaminectomy	++	++	Scaffold implantation	[61]
16	T9–12	Transverse resection of a spinal cord segment	++	+++	Investigation of the effect of autologous olfactory ensheathing cells on spinal cord regeneration	[11]
17	T10	Contusion	++	+++	Investigation of contusion injury	[23]
18	T10	Transverse resection of a spinal cord segment	++	+++	Investigation of myelination of injured nerve fibers and formation of a glial scar; study of functional recovery using neuronal stem cells	[41, 44]
19	T10–11	Chemical injury	++	+++	Investigation of magnetic field-driven migration of astrocytes to the injury site	[42]
21	T11	Electrostimulation	+++	++	Comparison of compensatory abilities in primates and rats in spinal cord injury	[1]
22	T11–12	Complete transection	+++	+++	Implantation of scaffolds; investigation of the effect of neuronal factor on axonal regeneration	[74]
23	L1–5	Transverse resection of a spinal cord segment	++	+++	Investigation of regeneration of motor neuron axons	[43]

*Severity: + – mild, ++ – moderate; +++ – pronounced.

## CONCLUSION

This review has described the main approaches to and features of SCI modeling in laboratory rats and demonstrated the use of biodegradable 3D scaffolds for restoring the functions of an injured spinal cord. However, each SCI model should be improved and adapted to the type and form of a new tested scaffold. The relationship between a quantitative recovery of axons and maintenance of the motor function after injury depends on the model type, material, and shape of the scaffold. Generalized data on the main experimental models of SCI in rats are presented in the Table.

The presented data, unfortunately, do not reflect the entire range of SCI models developed to date. Their number continues to increase. The advantages and disadvantages of each model should be considered in the context of its etiological and pathogenetic conformity to a human disease. Model adequacy is a key criterion for evaluating the possibility of extrapolating the findings to clinical practice. The question of to which extent the results obtained in rat biomodels can be extrapolated to humans is both of utmost importance and complexity in experimental animal modeling [95, 96]. The question of the adequacy of a given experimental biomodel to processes occurring in the human body remains open for most animal models.

## Abbreviations

SCI: spinal cord injury
C: cervical spine
T: thoracic spine
L: lumbar spine
